# Chinese SLE Treatment and Research Group Registry (CSTAR) XIV: the subjective well-being of patients with systemic lupus erythematosus

**DOI:** 10.3389/fmed.2022.984183

**Published:** 2022-09-20

**Authors:** Yue Shi, Dandan Bi, Yanhong Wang, Ruofan Li, Lijun Wu, Cheng Zhao, Zhenbiao Wu, Xinwang Duan, Jian Xu, Feng Zhan, Min Yang, Shengyun Liu, Qin Li, Shuo Zhang, Lingshan Liu, Jiuliang Zhao, Xinping Tian, Xinying Li, Qian Wang, Xiaofeng Zeng, CSTAR Co-authors

**Affiliations:** ^1^Department of Rheumatology and Clinical Immunology, Chinese Academy of Medical Sciences and Peking Union Medical College, National Clinical Research Center for Dermatologic and Immunologic Diseases (NCRC-DID), Ministry of Science and Technology, State Key Laboratory of Complex Severe and Rare Diseases, Peking Union Medical College Hospital (PUMCH), Key Laboratory of Rheumatology and Clinical Immunology, Ministry of Education, Beijing, China; ^2^CAS Key Laboratory of Mental Health, Institute of Psychology, Beijing, China; ^3^University of Chinese Academy of Sciences, Beijing, China; ^4^Department of Epidemiology and Biostatistics, School of Basic Medicine Peking Union Medical College, Institute of Basic Medical Sciences Chinese Academy of Medical Sciences, Beijing, China; ^5^Department of International Education, The Experimental High School Attached to Beijing Normal University, Beijing, China; ^6^Department of Rheumatology, The People’s Hospital of Xinjiang Autonomous, Urumqi, China; ^7^Department of Rheumatology, First Affiliated Hospital of Guangxi Medical University, Nanning, China; ^8^Department of Clinical Immunology and Rheumatology, Xijing Hospital Affiliated with The Fourth Military Medical University, Xi’an, China; ^9^Department of Rheumatology, The Second Affiliated Hospital of Nanchang University, Nanchang, China; ^10^Department of Rheumatology, First Affiliated Hospital of Kunming Medical University, Kunming, China; ^11^Department of Rheumatology, Hainan Provincial People’s Hospital, Haikou, China; ^12^Department of Rheumatology, Nanfang Hospital, Southern Medical University, Guangzhou, China; ^13^Department of Rheumatology, First Affiliated Hospital of Zhengzhou University, Zhengzhou, China; ^14^Department of Rheumatology, The First People’s Hospital of Yunnan Province, Kunming, China

**Keywords:** systemic lupus erythematosus, subjective well-being, life satisfaction, skin involvement, organ damage, quality of life

## Abstract

**Background:**

Systemic lupus erythematosus (SLE) can significantly influence patients’ quality of life and subjective well-being (SWB), but the relationships between clinical characteristics, SWB, and related psychological factors have been little studied.

**Objective:**

To measure SWB in patients with SLE and examine how major clinical determinants, emotional variables, and related positive factors affect SWB.

**Methods:**

Overall, 1,110 patients with SLE from the Chinese SLE Treatment and Research Group (CSTAR) and 198 age and gender-matched individuals from the general population without self-reported SLE were invited to complete questionnaires of SWB evaluated by the satisfaction with life scale (SWLS), emotional variables assessed by the patient health questionnaire-9 (PHQ-9), and general anxiety disorder-7 (GAD-7) and related positive factors assessed by the self-esteem scale (SES), general self-efficacy scale (GESE), and Connor-Davidson resilience scale (CD-RISC). The multivariate linear regression was used to examine the relationship between clinical manifestations and SWB.

**Results:**

Life satisfaction was significantly lower (*p* < 0.001) in patients with SLE than in the general population. Active skin involvement (OR = 0.923, 95% CI = 0.868–0.981, *p* < 0.05) was negatively associated with life satisfaction scores, and age at enrollment (OR = 1.160, 95% CI = 1.092–1.230, *p* < 0.001) were positively associated with life satisfaction scores in the multivariate regression model. The cumulative organ damage was significantly associated with depression (OR = 1.085, 95% CI = 1.022–1.153, *p* < 0.01) and the loss of self-esteem (OR = 1.067, 95% CI = 1.004–1.133, *p* < 0.05).

**Conclusion:**

SWB provides useful insight into the impact of SLE on psychological health and opportunities to improve quality of life and clinical care.

## Introduction

Systemic lupus erythematosus (SLE) is a chronic, progressive, autoimmune disease with complex clinical presentations ranging from mild arthralgia and skin rash to multiple organ involvement (1). In recent decades, survival among patients with SLE has improved and the treatment focus has shifted toward improving health-related quality of life (HRQoL) and long-term morbidity (2). Some disease-specific HRQoL instruments including Lupus Patient-Reported Outcome (LupusPRO), Lupus Quality of Life (LupusQoL), and Systemic Lupus Erythematosus-Specific Quality of Life Questionnaire (SLEQOL) are widely administered in clinical practice and clinical studies to better capture symptoms or issues that are specific to SLE than generic instruments (3, 4). HRQOL and subjective symptoms, including anxiety, fatigue, low self-esteem, and cognitive impairment are central to perspectives of adults living with SLE and have been found to be associated with subsequently greater cumulative organ damage (5, 6). Recently, subjective well-being (SWB) has gained increasing attention for mental health-related evaluation and has been referred to as an indicator of psychological adjustment (7, 8).

Life satisfaction is one of the major components of SWB. Life satisfaction is defined as a cognitive judgment process of personal life evaluation and is generally measured by the satisfaction with life scale (SWLS) (9). Studies have suggested that life satisfaction correlates with HRQoL in patients with SLE (10). Furthermore, SWB is affected by emotional variables including depression and anxiety (11), and related positive factors, such as self-esteem, self-efficacy, and resilience (12). These emotional variables and related positive factors can perform a mediating role in the relationship between disease burden and SWB (13, 14). However, no previous study has examined the association between SWB and these psychological factors in patients with SLE.

Poor self-reported mental health and life dissatisfaction have been reported to be associated with increased mortality and serve as general health risk indicators (15). The experience of patients with SLE related to disease activity and organ damage may predict a worse HRQoL, especially in the components of mental health, physical capability, and planning (16). However, the influence of disease activity and accumulated damage on SWB is still unknown. In 2016, an international expert panel (the definition of Remission in SLE, DORIS project) proposed that remission or the lupus low disease activity state (LDAS) could be potential treating target for patients with SLE; but if such cannot be reached, the lupus low disease activity state (LDAS) could be another potential treat to target goal (17). Despite growing evidence that being in remission or LDAS is associated with a better HRQoL in SLE (16, 18), few studies have evaluated the relationship between remission/LDAS and psychological well-being. This study aimed to evaluate SWB and related psychological factors of patients with SLE and to study their main clinical determinants and the impact on life satisfaction as an indicator of SWB.

## Materials and methods

### Samples and procedures

A total of 1,110 patients with SLE were enrolled in the Chinese SLE Treatment and Research Group (CSTAR) online registry covering 21 rheumatologic clinical centers in 15 provinces in China from 8 March 2011 to 7 June 2019. All the patients fulfilled the Systemic Lupus International Collaborating Clinics (SLICC) (19) classification criteria for SLE and had at least two clinical visits. Data were collected through the CSTAR online registry, including demographic characteristics, clinical features, laboratory examinations, disease activity evaluated by the SLE disease activity index 2000 (SLEDAI-2K) (20), and systemic lupus international collaborating clinics/ACR damage index (SLICC/SDI) and treatments. The LLDAS was defined as follows: (1) SLEDAI-2K ≤ 4 with no scores for the renal, central nervous system, cardiopulmonary, vasculitis, fever, hemolytic anemia, or gastrointestinal activity; (2) No increase in any SLEDAI-2K component since the previous visit; (3) Physician global assessment (PGA) ≤ 1; and (4) stable immunosuppressants or biological immunomodulators and less than 7.5 mg/day prednisone (or equivalent) (21). The definition of remission is as follows: (1) clinical SLEDAI-2K = 0; (2) PGA < 0.5; (3) stable immunosuppressants or biological immunomodulators and less than 5 mg/day prednisone (or equivalent)(17). We recruited 198 age- and gender-matched controls through the Wenjuanxing platform among the general social group who self-declared that they were not suffering from SLE or other diseases.

### Psychological measures

SWB was assessed by life satisfaction scores and related psychological factors including self-esteem, self-efficacy, resilience, depression, and anxiety. Life satisfaction was evaluated by the SWLS (9) containing five items rated on a 7-point Likert scale (1 = strongly disagree; 7 = strongly agree). Higher scores reflect higher life satisfaction. Emotional variables including depression and anxiety were assessed by the patient health questionnaire-9 (PHQ-9) and general anxiety disorder-7 (GAD-7) scales. Related positive factors, including self-esteem, self-efficacy, and resilience were assessed by the self-esteem scale (SES), general self-efficacy scale (GESE), and Connor-Davidson resilience scale (CD-RISC), respectively. The SES includes 10 items (e.g., “I feel that I am a person of worth, at least on an equal plane with others”) on a 4-point scale (22). Higher scores reflect lower self-esteem. The GESE is a widely internationally used scale containing 10 items, for example, “I am confident that I can deal with anything unexpected” (23). This scale has a scoring system ranging from 0 to 3. Higher scores reflect higher self-efficacy. Resilience was measured by the 25-item CD-RISC, which has been well validated in the Chinese population (24). This scale is a 5-point Likert scale (0 = not true at all, 4 = true nearly all of the time), with a higher score indicating greater resilience. The reliability of all psychological measurements used in our research has been validated among Chinese general population.

### Statistical analysis

The Shapiro–Wilk test was used to test for a normal distribution of continuous variables. The continuous variables were summarized as the means and standard deviation (SD) if normally distributed, or as the median and interquartile range (IQR) if they had a skewed distribution. The *t*-tests and χ2-tests were conducted for univariate analysis. The Pearson and Spearman tests were used for linear correlation between psychological measures. A linear regression model was used for multivariate analysis, with SWB and related psychological factors as dependent variables and age, sex, LDAS, and the variables which were significantly associated within univariate analysis as independent variables. Statistical analyses were performed using R version 3.6 (R Core Team, Vienna, Austria) and SPSS 25.0 (IBM, Armonk, NY, USA).

### Ethics statement

Informed consent was obtained from all participants. This study was approved by the Medical Ethics Committee of Peking Union Medical College Hospital, Chinese Academy of Medical Sciences.

## Results

Participants’ clinical and psychological characteristics are shown in Table 1. A total of 1,062 (95.7%) were females and lupus duration at recruitment was 6.00 (IQR 2.00–10.00) years. The compliance with the Systemic Lupus International Collaborating Clinics (SLICC) 2012 classification criteria is shown in Supplementary Table 1. The SLEDAI indices were analyzed: musculoskeletal involvement (4.9%), renal involvement (11.7%), skin involvement (9.1%), etc. In total, 105 (9.5%) patients achieved remission, and 283 (25.5%) patients achieved LDAS. Additional data on treatments are shown in Supplementary Table 2. Life satisfaction was 21.13 ± 6.75 among patients with SLE and 23.76 ± 5.41 in the general population (*p* < 0.001). Depression of patients with SLE was 7.47 ± 5.31, in contrast to 5.20 ± 3.90 in the general population (*p* < 0.001). The SWB and related psychological factors questionnaires all demonstrated good reliability (Cronbach’s α = 0.857–0.931).

**TABLE 1:** Comparison of clinical and psychological characteristics of 1,110 patients with SLE and 198 individuals from the general population without self-reported SLE.

Variable	SLE (*n* = 1,110)	Non-SLE (*n* = 198)	*P*-value	Cronbach α
Gender, female	1,062 (95.7%)	183 (92.4%)	0.074	−
Age (years)	34.02 ± 9.49	34.71 ± 3.69	0.076	−
Duration of SLE (years)	6.00 (2.00, 10.00)	−	−	−
SLEDAI	2.00 (0.00, 4.00)	−	−	−
Musculoskeletal	54 (4.9%)	−	−	−
Renal	130 (11.7%)	−	−	−
Skin	101 (9.1%)	−	−	−
Hematologic	108 (9.7%)	−	−	−
Neuropsychiatric	22 (2.0%)	−	−	−
Serositis	15 (1.4%)	−	−	−
SDI ≥ 1	290 (26.1%)	−	−	−
Cumulative organ involvement				
Musculoskeletal	603 (54.2%)	−	−	−
Renal	475 (42.8%)	−	−	−
Skin	660 (59.5%)	−	−	−
Hematologic	529 (47.7%)	−	−	−
Neuropsychiatric	78 (7.0%)	−	−	−
Serositis	117 (10.5%)	−	−	−
SDI ≥ 1	294 (26.4%)	−	−	−
LDAS	283 (25.5%)	−	−	−
Remission	105 (9.5%)	−	−	−
Treatment				
Glucocorticoids	1,016 (92.3%)	−	−	−
Immunosuppressive drugs	1,100 (99.1%)	−	−	−
**Psychological measures**				
Life satisfaction	21.13 ± 6.75	23.76 ± 5.41	<0.001	0.875
Loss of self-esteem	22.06 ± 4.12	18.91 ± 4.00	<0.001	0.863
Self-efficacy	24.30 ± 6.15	26.82 ± 4.28	<0.001	0.910
Resilience	56.26 ± 15.29	65.48 ± 11.20	<0.001	0.931
Depression	7.47 ± 5.31	5.20 ± 3.90	<0.001	0.857
Anxiety	5.64 ± 4.87	4.35 ± 3.53	0.005	0.915

Data given as mean ± SD, median (IQR) or n (%). SLE, systemic lupus erythematosus; Non-SLE, general population individuals without self-reported SLE; SLEDAI, systemic lupus erythematosus disease activity index; SDI, systemic lupus international collaborating clinics/American college of rheumatology damage index; LDAS, low disease activity status; Life satisfaction, SWLS (satisfaction with life scale) scores; Self-esteem, SES (Self-Esteem Scale) scores; Self-efficacy, GESE (General Self-Efficacy Scale) scores; Resilience, CD-RISC (Connor-Davidson Resilience Scale) scores; Depression, PHQ (Patient Health Questionnaire) scores; Anxiety, GAD (General Anxiety Disorder) scores.

**FIGURE 1:**
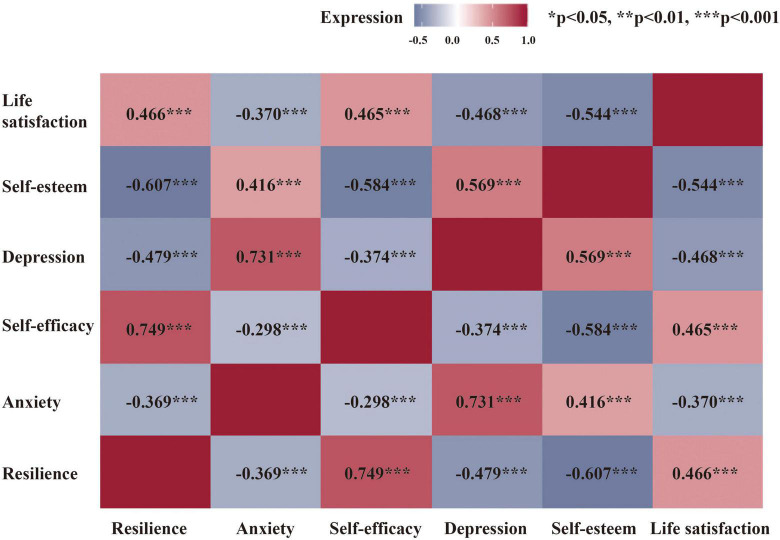
Correlation between subjective well-being and related psychological factors. For abbreviations, see the previous table.

As shown in Figure 1, we observed significant correlations between the life satisfaction scores and the scores of all the related psychological factors (loss of self-esteem, depression, self-efficacy, anxiety, and resilience). Depression (*r* = −0.468, *p* < 0.001) and the loss of self-esteem (*r* = −0.544, *p* < 0.001) appeared to be negatively correlated with life satisfaction scores.

The clinical determinants of SWB and related psychological factors in the multivariate linear regression model are depicted in Table 2. Active skin involvement (OR = 0.923, 95% CI = 0.868–0.981, *p* < 0.05) had negative predictive effects on life satisfaction scores. Otherwise, age at enrollment (OR = 1.160, 95% CI = 1.092–1.230, *p* < 0.001) had positive predictive effects on life satisfaction scores. Higher damage index predicted higher depression scores (OR = 1.085, 95% CI = 1.022–1.153, *p* < 0.01) and the loss of self-esteem (OR = 1.067, 95%CI = 1.004–1.133, *p* < 0.05). Moreover, age at enrollment was strong predictors of less self-esteem loss (OR = 0.914, 95% CI = 0.861–0.971, *p* < 0.01) and lower depression scores (OR = 0.922, 95% CI = 0.868–0.979, *p* < 0.01).

**TABLE 2:** Multivariate linear analysis of the major determinants of subjective well-being and related psychological factors.

	Life satisfaction		Resilience		Loss of self-esteem		Depression	
				
	OR (95% CI)	*P*	OR (95% CI)	*P*	OR (95% CI)	*P*	OR (95% CI)	*P*
Age at enrollment	1.160 (1.092, 1.230)	<0.001	0.950 (0.893, 1.012)	0.152	0.914 (0.861, 0.971)	0.004	0.922 (0.868, 0.979)	0.008
LDA	0.983 (0.924, 1.045)	0.583	0.967 (0.908, 1.027)	0.274	0.969 (0.912, 1.029)	0.305	0.978 (0.919, 1.041)	0.483
SDI	−	−	−	−	1.067 (1.004, 1.133)	0.036	1.085 (1.022, 1.153)	0.007
Active skin involvement	0.923 (0.868, 0.981)	0.010	0.950 (0.893, 1.012)	0.112	−	−	1.055 (0.989, 1.126)	0.102
Active renal involvement	0.923 (0.891, 1.006)	0.077	−	−	−	−	−	−
Active musculoskeletal involvement	−	−	−	−	−	−	1.065 (0.999, 1.135)	0.055
Active hematologic involvement	−	−	−	−	−	−	1.050 (0.988, 1.116)	0.113
Adjusted *R*^2^	0.032		0.007		0.009		0.022	

After adjusting for gender. OR, odds ratio; CI, confidence interval (for other abbreviations, see the previous table).

## Discussion

This is the first study reporting SWB and its related psychological factors among Chinese patients with SLE and identifying its relationship with active disease manifestations and damage index. Our results show that life satisfaction is significantly impaired in patients with SLE compared with the general population. Active skin involvement and age are significantly associated with life satisfaction in the linear regression model.

Low life satisfaction scores obtained by investigated patients, in a significant way, confirm how serious is the psychological problem in SLE. Similar life satisfaction results were presented in patients with cancer (7). Although many studies addressed the high prevalence of depression and anxiety in patients with SLE (25), little attention has been paid to the patient’s positive attitude which seems to be a crucial determinant of therapeutic adherence (26, 27). People may experience very low levels of SWB even in the absence of overt depression or anxiety. Moreover, SWB and related psychological factors contribute substantially to low HRQoL in patients with SLE (10, 28). Some disease-specific HRQoL instruments may also help recognize major mental health disorders promptly (29). However, the relationship between SWB and these disease-specific HRQoL in patients with SLE needs to be further evaluated. In routine care visits or mental health studies of patients with SLE, SWB should be considered as a supplementary assessment of health status to achieve a more holistic assessment of patients’ lives and optimize lupus care.

Skin injury is the second most common manifestation of patients with SLE after renal impairment (30). Patients with skin injury tend to have a higher risk for mental comorbidities and experience lower levels of happiness (31). The rashes and lesions on the skin of patients with SLE could cause severe physical changes in appearance. Individuals with body image concerns reported more psychosocial issues, including impaired psychosocial functioning (32). SWB in patients with skin diseases is found to be linked to better health outcomes, thus integrating SWB into the treatment of the diseases seems a promising approach (31, 33). Moreover, some scholars stated that emotional stress seems to upregulate inflammation and could aggravate some chronic inflammatory diseases (34). Although the most serious problems of SLE are often attributable to internal organ damage, patients with skin involvement deserve more attention in mental health evaluation and require a new type of treatment strategy integrating wellbeing improvement into the target.

Decades of psychological research revealed that external factors such as demographic characteristics including marital status, income, and educational level only had a modest impact on SWB (35). Thus, a lot of work has focused on the significant role of internal personality on SWB. Personality dispositions such as self-esteem were significantly associated with life satisfaction (36), which was consistent with our finding in SLE. Loss of self-esteem was reported to be prevalent in patients with SLE (6) and associated with greater cumulative organ damage (5). Physician-measured damage score is the focus in routine clinical practice as a poor prognostic sign and a predictor of mortality (37). Accumulated damage was associated with quality of life due to activity limitation in patients with SLE with skin and joint involvement (38). In our study, SDI was associated with depression and the loss of self-esteem. Thus, for patients with SLE with a greater physician-measured organ damage index, qualitative research can provide a comprehensive assessment of experiences and beliefs of SLE from the patient’s perspective. Moreover, psychotherapy (39) and some lupus self-management programs (40) have already been studied as interventions in clinical trials, which showed improvement in patients’ health outcomes. Therefore, future research should consider a combination of medication and psychological interventions as a whole, especially for patients with more organ damage.

In the wake of the principle of treating-to-target (T2T) in rheumatoid arthritis, remission and LDAS have been proposed as desirable therapeutic goals for patients with SLE (2, 21, 41). Emerging studies have demonstrated that the attainment of remission/LDAS was associated with improved outcomes in SLE, including lower damage accrual, lower probability of flares, and a better HRQoL (42). Regarding HRQoL, being on remission/LDAS was reported to predict higher scores in the components of physical health, but not in the components of mental health (16, 18, 42). The data from our study also indicated a lack of associations between remission/LDAS and SWB, probably because the mental domains are influenced by more complicated factors such as demographic characteristics, personality, and culture except for SLE-related factors involved in the definition of remission/LDAS at present. Thus, mental health evaluation and adjustment should be considered to incorporate into the treatment target of SLE. However, Heijke et al. reported that disease duration might affect patient-reported outcome measures (43). The results of the relationship between clinical determinants and SWB in patients with SLE duration of 1 year or less are shown in Supplementary Table 3. In patients with recent-onset SLE, LDAS appears to be associated with the related psychological factors of SWB. The interplay between remission/LDAS and patient-reported outcome measures might be influenced by SLE disease duration. This suggests that the mental health evaluation of patients with recent-onset SLE needs more attention.

The limitation of our study is the absence of quality-of-life assessments. The relationship between HRQoL and SWB in patients with SLE needs to be further evaluated. Second, many confounding factors such as socioeconomic status and education level may affect SWB, which could cause biases in our results. Third, patients with more active disease or specific active organ involvement should be considered in future research on the relationship between disease activity and SWB. Fourth, we recruited controls through the Wenjuanxing platform among the general population without self-reported SLE, which might have caused biases. Moreover, the effect of steroids, antimalarials, and immunosuppressants including the dosage and mode of administration on SWB warrant further investigation. SLE is quite heterogeneous, so including patients with different clinical manifestations in different percentages might cause more heterogeneity in the results when compared to the general population.

## Conclusion

In conclusion, SLE is a multisystem disorder associated with poor SWB status. Active skin involvement and higher organ damage index are the major clinical determinants of SWB and its related psychological factors in patients with SLE. SWB may be a potential psychological outcome in clinical trials and should be considered when developing therapeutic targets for SLE management.

## Data availability statement

The data analyzed in this study is subject to the following licenses/restrictions: The dataset used and/or analyzed during the current study are available from the corresponding author on reasonable request. Requests to access these datasets should be directed to the corresponding author.

## Ethics statement

The studies involving human participants were reviewed and approved by the Ethics Committee of Peking Union Medical College Hospital. All participants gave informed consent to participate in the study before taking part. The patients/participants provided their written informed consent to participate in this study.

## Author contributions

XL and QW were the guarantor and took responsibility for the integrity of the work. XL, QW, XT, and XZ conceived and designed the study. YS, DB, and YW performed the data analysis and drafted the manuscript. All authors critically revised the manuscript and approved the final manuscript, contributed to the acquisition, and interpretation of the data.
