# Supplementary material for: Chinese SLE Treatment and Research Group Registry (CSTAR) XIV: the subjective well-being of patients with systemic lupus erythematosus
Source: Front Med (Lausanne). 2022 Sep 20;9:984183. doi: 10.3389/fmed.2022.984183 (PMC9531862; doi:10.3389/fmed.2022.984183)
Supplement: Supplementary file 1 [file Table_1.pdf]

Supplementary Table 1. The Systemic Lupus International Collaborating Clinics (SLICC) 2012 classification criteria in 1110 patients with SLE.

	**SLE(n=1110)**
**SLICC clinical criteria**	
Acute/subacute cutaneous rashes	487 (44.3%)
Chronic cutaneous rashes	173 (15.7%)
Oral/nasal ulcers	210 (19.1%)
Alopecia	509 (46.3%)
Arthritis	602 (54.7%)
Serositis	117 (10.6%)
Renal	475 (43.2%)
Neurological	71 (6.5%)
Anaemia	77 (7.0%)
Leucopenia/lymphopenia	354 (32.2%)
Thrombocytopenia	211 (19.2%)
**SLICC immunological criteria**	
ANA	1098 (99.8%)
Anti-dsDNA	816 (74.2%)
Anti-Sm	413 (37.5%)
Antiphospholipid	271 (24.6%)
Complement	806 (73.3%)
Coombs	226 (20.5%)

Data are presented as n (%).

Supplementary Table 2. Therapeutic information of SLE patients.

	**SLE(n=1110)**
**Corticosteroids**	
Prednisone	702 (63.8%)
Methylprednisolone	363 (33.0%)
**DMARDs**	
Hydroxychloroquine	988 (89.9%)
Azathioprine	143 (13.0%)
Methotrexate	155 (14.1%)
Leflunomide	111 (10.1%)
**Other immunosuppressants**	
Mycophenolate	330 (30.0%)
Cyclosporine	72 (6.6%)
Cyclophosphamide	171 (15.6%)
Tacrolimus	194 (17.6%)
**Comedications**	
Antihypertensives and diuretics	240 (21.8%)
Platelet antiaggregants	197 (17.9%)

Data are presented as n (%).

Abbreviations: DMARDs, synthetic disease-modifying antirheumatic drugs.

Supplementary Table 3. Multivariate linear analysis of the major determinants of subjective well-being and related psychological factors in patients with SLE duration of one year or less (n=174).

	Life satisfaction		Resilience		Self-esteem		Depression	
	**OR (95% CI)**	**P**	**OR (95% CI)**	**P**	**OR (95% CI)**	**P**	**OR (95% CI)**	**P**
**Age at enrollment**	1.160 (1.092,1.230)	< 0.001	0.950 (0.893,1.012)	0.152	0.914 (0.861,0.971)	0.004	0.922 (0.868,0.979)	0.008
**LDAS**	0.983 (0.924,1.045)	0.583	0.967 (0.908,1.027)	0.274	0.969 (0.912,1.029)	0.305	0.978 (0.919,1.041)	0.483
**SDI**	-	-	-	-	1.067 (1.004,1.133)	0.036	1.085 (1.022,1.153)	0.007
**Active skin involvement**	0.923 (0.868, 0.981)	0.010	0.950 (0.893,1.012)	0.112	-	-	1.055 (0.989,1.126)	0.102
**Active renal involvement**	0.923 (0.891,1.006)	0.077	-	-	-	-	-	-
**Active musculoskeletal involvement**	-	-	-	-	-	-	1.065 (0.999, 1.135)	0.055
**Active hematologic involvement**	-	-	-	-	-	-	1.050 (0.988, 1.116)	0.113
**Adjusted R^2**	0.032		0.007		0.009		0.022	

After adjusting for gender.

Abbreviations: OR, odds ratio; CI, confidence interval; LDAS, low disease activity status; SDI, systemic lupus international collaborating clinics/American college of rheumatology damage index.
